# Editorial: Spirituality and positive psychology

**DOI:** 10.3389/fpsyg.2023.1202359

**Published:** 2023-05-19

**Authors:** Nicolas Roussiau, Christian R. Bellehumeur, Cynthia Bilodeau, Oscar Navarro, Nathalie Bailly, Caesar Tapia-Fonllem, Llewellyn Van Zyl, Elise Renard

**Affiliations:** ^1^Université de Nantes, Nantes, France; ^2^University of Ottawa, Ottawa, ON, Canada; ^3^University of Nîmes, Nîmes, France; ^4^Université de Tours, Tours, France; ^5^University of Sonora, Hermosillo, Mexico; ^6^University of Eindhoven, Human Performance Management Group, Eindhoven, Netherlands; ^7^Optentia Research Unit, North-West University (Vaal Triangle Campus), Vanderbijlpark, South Africa; ^8^Department of Human Resource Management, University of Twente, Enschede, Netherlands; ^9^Department of Social Psychology, Goethe University, Frankfurt am Main, Germany; ^10^Laboratoire LPPL, Université de Nantes, Chemin de la Censive-du-Tertre, Nantes, France

**Keywords:** positive psychology, hope, nature, spirituality, questionnaires

The emergence of positive psychology in the broader field of psychology has resulted in an epistemological and theoretical revolution and paradigm shift that have, in turn, had numerous implications on research orientations (Seligman, 1999; Seligman and Csikszentmihalyi, 2000; Peterson and Seligman, 2004). This shift continues to lead psychological research to unexplored areas and to topics that, until now, have been singularly absent or marginalized. This is the case for spirituality, which, until recently, was dismissed to the realm of religious or mystical belief and consequently, has not been the subject of systematic scientific study in many disciplines of psychology.

Positive psychology rapidly highlighted the importance of addressing this topic in its lines of research, as illustrated by the book “Handbook of positive psychology” by Snyder and Lopez (2002), in which several authors develop spirituality in their research (e.g., Pargament and Mahoney, 2002). This growing interest is more and more visible in scientific works; examples include “Positive psychology and spirituality in psychotherapy” by Bellehumeur and Malette (2019) and “Handbook of Positive Psychology, Religion, and Spirituality” by Davis et al. (2023). Spirituality as a topic now occupies a central position alongside positive psychology. The time has come to address spirituality in its own right.

This Research Topic, which brings together spirituality and positive psychology, focuses on powerful topics such as the relationship with “nature” and “hope,” but it also addresses the question of measuring spirituality using scales, and the links between spirituality and the study of character in positive psychology.

In their article on the integration of positive psychology and spirituality in the context of climate change, Bellehumeur et al. explore the effects of climate change as an issue and its impact on stress and health. They argue the idea that positive psychology can benefit from the integration of spirituality to better support people's wellbeing. Their epistemological presentation presents the progressive integration of spirituality into positive psychology from the first to the third wave. Let us consider the sublime, wonder, and the appreciation of beauty, powerful topics that enlighten our relationship with nature. In their field research in the Arctic wilderness, Løvoll and Sæther demonstrate that feelings experienced when faced with natural beauty can be life-transforming and may promote the awareness of nature as an existential value. Their results support the links between wonder, spirituality and wellbeing.

Hope is the second highlight of this Research Topic. In their article, Wang et al. examine the unexplored link between childhood socioeconomic status and subjective wellbeing in adulthood using data from a field survey of rural residents of poor areas in China. The authors show that hope and a sense of control can mitigate the negative effects of experienced childhood poverty on subjective wellbeing in adulthood. Laranjeira et al. revisit the question of hope with an ambitious goal: to promote hope as spiritual care. Experiencing hope conveys peace and security in the present, introducing spirituality and hope into palliative care programs is a humanistic approach that combines positive psychology and spirituality.

The very particular topic that is spirituality leads to stances that sometimes question the process of quantification. However, refusing to measure is to disqualify spirituality from a part of science. The following articles propose several scales for evaluating different aspects of spirituality. In their article, following up on James Fowler's work on the development of faith, Mallery and Mallery present a scale to assess the search for and the creation of meaning. Armstrong and Potter develop a first, brief, self-assessment to measure adaptation to the COVID-19 pandemic. Wüthrich-Grossenbacher et al. present the Religious and Spiritual Struggles (RSS) scale with an intercultural perspective that measures the internal and external conflicts with religion and spirituality experienced among Zimbabwean adolescents living with HIV.

Finally, Ford et al. discuss the links between spirituality and the study of character in positive psychology using different methods to better understand latent profiles such as the spiritual character.

To conclude this introduction, we would like to emphasize two points that put this special Research Topic into perspective. First, is the popular culture which views spirituality as being positive and the source of wellbeing. This caricatured view is evident, for example, in the opposition that views religion as negative and spirituality as positive, a Manichean view that has been noted many times (Zinnbauer et al., 1999). We must not forget that the spiritual process (religious or not) can trigger real suffering (Pargament et al., 2004; Jones et al., 2015) and if we must agree on a definition of spirituality, then ethics and values should be the foundation of a spirituality that is both humanistic and positive. Failure to consider the darker sides of spirituality could undermine its study and reinforce criticism that brings into question the impartiality of certain researchers.

The second point relates to the focus on a strictly positive vision of existence, which prevents us from grasping the complexity of human life. From a dialectical perspective, negative emotions can serve the adaptive functions of survival and self-fulfillment. As it happens, these existential questions are easily anchored to spirituality whose essential function is a search for meaning. The existential perspective of positive psychology (Wong, 2010), aims at personal fulfillment, while accepting the potential confrontations that arise from human reality that is both positive and negative. The goal is to transcend the dark sides of one's existence in order to develop personal strengths, because out of existential suffering can emerge a profound sense of meaning for one's existence. These precautions are necessary to avoid falling into a caricatured vision of both positive psychology and spirituality.

## Author contributions

NR acted as the co-ordinator of the Research Topic and wrote the first draft of the editorial. CRB, CB, NB, ON, CT-F, LVZ, and ER made significant intellectual contributions to the Research Topic and provided input on the editorial. All authors contributed to the article and approved the submitted version.
